# Plasma Cytokines Level and Spinal Cord MRI Predict Clinical Outcome in a Rat Glial Scar Cryoinjury Model

**DOI:** 10.3390/biomedicines10102345

**Published:** 2022-09-21

**Authors:** Georgii B. Telegin, Aleksandr S. Chernov, Alexey N. Minakov, Maksim V. Rodionov, Vitaly A. Kazakov, Viktor A. Palikov, Irina P. Balmasova, Dmitry S. Asyutin, Yuri M. Poluektov, Nikolay A. Konovalov, Anna A. Kudriaeva, Aldo Spallone, Alexander G. Gabibov, Alexey A. Belogurov

**Affiliations:** 1 Branch of Shemyakin and Ovchinnikov Institute of Bioorganic Chemistry, Russian Academy of Sciences, 142290 Pushchino, Russia; 2 Medical Radiological Research Center (MRRC) Named after A.F. Tsyb, Branch of the National Medical Radiological Research Center, Ministry of Health of Russian Federation, 249031 Moscow, Russia; 3 Ministry of Health of Russian Federation, Evdokimov Moscow State University of Medicine and Dentistry, 127473 Moscow, Russia; 4 N.N. Burdenko National Scientific and Practical Center for Neurosurgery, Ministry of Health of Russian Federation, 125047 Moscow, Russia; 5 Shemyakin and Ovchinnikov Institute of Bioorganic Chemistry, Russian Academy of Sciences, 117997 Moscow, Russia; 6 NCL—Neurological Centre of Latium, 00178 Rome, Italy

**Keywords:** spinal cord injury, cryoapplication, glial scar, SD rats, magnetic resonance imaging, computed tomography, multiplex, cytokines, chemokines

## Abstract

Traumatic injury of the spinal cord is still one of the most challenging problems in the neurosurgical practice. Despite a long history of implementation of translational medicine in the field of spinal cord injury (SCI), it remains one of the most frequent causes of human disability and a critical situation for world healthcare systems. Here, we used our rat model of the of unilateral controlled SCI induced by a cryoinjury, which consistently reproduces glial scarring and posttraumatic cyst formation, and specifically evaluated histological, bioimaging and cytokine data. We propose a 10-grade scoring scale, which can objectively estimate the extent of damage of the experimental SCI according to the magnetic resonance imaging (MRI) results. It provides a homogeneous and reliable visual control of the dynamics of the posttraumatic processes, which makes it possible to clearly distinguish the extent of early damage, the formation of glial scars and the development of posttraumatic syringomyelic cysts. The concentration of cytokines and chemokines in the plasma following the experimental SCI increased up to two orders of magnitude in comparison with intact animals, suggesting that a traumatic injury of the spinal cord was accompanied by a remarkable cytokine storm. Our data suggested that the levels of IL-1α, IL-1β, TNFα, GRO/KC, G-CSF, IFNγ and IL-13 may be considered as a reliable prognostic index for SCI. Finally, we demonstrated that MRI together with plasma cytokines level directly correlated and reliably predicted the clinical outcome following SCI. The present study brings novel noninvasive and intravital methods for the evaluation of the therapeutic efficacy of SCI treatment protocols, which may be easily translated into the clinical practice.

## 1. Introduction

Despite the enormous efforts of clinicians all over the world, a spinal cord injury (SCI) remains among the most critical problems in modern neurosurgery; thus, the social and economic impacts of this medical challenge cannot be overestimated. Epidemiological studies in developed countries indicate an SCI incidence of 4–6 cases per 100,000 inhabitants per year with serious long-term sequelae for the patients and, as a consequence, a tremendous impact on society [1]. Early decompressive surgery, though strongly recommended, did not show a significant impact on the long-term outcomes in SCI patients [2]. Therefore, SCI was considered as a hopeless situation until the introduction of stem cell therapy [3], which gave hope for curing traumatic SCI patients. Unfortunately, several proposed therapeutic modalities, which demonstrated promising results in experimental models, failed to show their efficacy in clinical trials. Very recently, the epidural stimulation of the lumbosacral spine has been proposed with the idea of overcoming the interruption of physiological neural signal transmission by bypassing the traumatized spinal segment [4]. Nonetheless, this method has shown promising clinical results only in thoracic cord trauma patients, who represent a minor cohort of individuals with SCI [5]. Concluding, the elaboration of novel approaches in SCI therapy requires the evaluation of complex pathophysiological processes following SCI.

One of the major obstacles to spinal cord regeneration following trauma is the formation of glial scarring, which impedes the proper growth of axons and myelinization across the traumatized cord segment [6]. Thus, reducing the posttraumatic scarring process could be a fundamental step for improving the proper regrowth of axons and clinical recovery [7]. Local neuroinflammation triggered by cytokines and chemokines may play a crucial negative role in the pathological processes following spinal trauma [8,9]. Thus, the examination of pro- and anti-inflammatory cytokine cascades during SCI might suggest a novel therapeutic strategy [10,11].

Here, we used a previously described, highly reproducible rodent model of unilateral controlled spinal cord trauma induced by a cryoinjury [12,13]. We studied specifically the time course of the posttraumatic process with magnetic resonance imaging (MRI) and the concentration of cytokine plasma and colony-stimulating factors as related to SCI clinical outcome.

## 2. Materials and Methods

### 2.1. Laboratory Animals

Male SD rats of SPF category with an average weight (±SD) of 398.9 (±10.4) g were used in this study. All animals were housed under standard conditions in the Animal Breeding Facility of BIBCh, RAS (the Unique Research Unit Bio-Model of the IBCh, RAS; the Bioresource Collection—Collection of SPF-Laboratory Rodents for Fundamental, Biomedical and Pharmacological Studies), which has an international accreditation AAALACi. All manipulations with the animals were approved by the IACUC of the BIBCh RAS (protocol no. 756/21 of 01.10.21).

### 2.2. Surgical Approach to Spinal Trauma

All surgical manipulations were performed in the sterile bloc of the “Animal Breeding Facility” by certified veterinarian surgeons and in accordance with the recommendations from the local ethical committee (protocol no. 756/21 of 01.10.21). The detailed surgical technique for the induction of the local cryospinal injury was described previously [12]. Briefly, a unilateral laminectomy of the Th13 vertebra was performed using a dental drill. Cryoinjury was induced with a thermally conductive assembly of an originally designed cryoprobe applied to the spinal cord through the dura mater. The exposure to the cryoprobe lasted for 1 min. In the contact area, the local temperature reached –20 °C. Intact animals without unilateral laminectomy of the Th13 vertebra were used as a control.

### 2.3. Treadmill Exercise Test

The running capacity of the animals was evaluated under blind conditions by examining the maximum achieved speed on a moving treadmill. Functional physiological tests were performed at days 7, 14, 21, 30 and 60 after trauma modeling, utilizing Animal Treadmill “Exer 3/6” (Columbus Instruments, Columbus, OH, USA). The angle of inclination of the treadmill was 0°. The set frequency of electric shock exposure was 2 Hz and the intensity was 1 mA. When the treadmill was turned off (and the speed set to 0 m/min), the rat was placed on a separate treadmill. After that, the current supply to the corresponding grid was turned on. The rats examined the treadmill before receiving the first electric shock. An introductory period of 1–2 min with an electric stimulus turned on was enough for animals to avoid contact with a metal grid with electric current during the test. Then, the movement of the track was started with an acceleration of 1 m/min every 5 s. The maximum speed was set at 50 m per minute. The test was continued until an animal reached a maximum speed of 50 m per minute or lost the ability to continue moving on a treadmill. This was defined as spending 10 s or more on the grid without a possibility for the rat to reengage in running. The primary aim was to delineate the maximum achieved speed on the moving treadmill, which was recorded for each animal and compared between groups using a Mann–Whitney test.

### 2.4. Magnetic Resonance Imaging of the Spine

MRI scans of the rat spinal cord were performed at day 1 (about 30 min after injury), and 7, 14, 21, 30 and 60 days after the cryoinjury, using a 7 T MRS*DRYMAG 7017PW device (MRSolutions, Guildford, Surrey, UK), with a universal transmit–receive RF coil for rats, with an inner diameter of 65 mm. During the imaging procedure, the animals were anesthetized with a 3% isoflurane in air and maintained in a specialized bed at a temperature of +37 °C; a respiratory gating function was used.

Standard FSE (fast spin echo) pulse sequences for acquiring T1- and T2-weighted images of caudal thoracic and cranial lumbar segments of the spine were used. The 0.5 mm slices without gaps were oriented in the axial plane, and the FOV used was 60 mm (rectangular). T1-weighted images scan parameters were: effective TE, 11 ms; TR, 1000 ms; echo train number, 4; echo-spacing, 11 ms; and number of averages, 3. T2-weighted images scan parameters were: effective TE, 45 ms; TR, 4000 ms; echo train number, 7; echo-spacing, 15 ms; and number of averages, 3.

The images acquired were processed using VivoQuant software (Invicro, London, UK). Regions of interest were determined as hypointense and hyperintense focal areas located at the level of Th13 spine on the right side. The segmentation of those was performed using a semiautomatic threshold mode to determine their volume in mm^3^ and their dimensionless ratio (VHyper/VHypo). The scoring scale was defined for a straightforward visual assessment of the severity of spinal cord injury (Table 1).

### 2.5. Computed Tomography Scans of the Spine

CT scans with contrast enhancement “Omnipaque-240” (Iohexol, GE Healthcare AS, Oslo, Norway) were performed at day 30 after the experimental spinal cord injury, with a iodine dose of 36 mg per injection. Before the injection, rats were sedated with a short-acting hypnotic agent (propofol, Hana Pharmaceutical Co. Ltd., Seoul, South Korea) via an intravenous bolus into the lateral tail vein at a dose of 20 mg/kg. The injection of contrast agent was carried out into the cisterna magna at a speed of 100 μL/s, with a 1 mL syringe armed with a G29 needle. Immediately after the injection, a CT scan of the spine was carried out using a CT scanner MRS*CT/PET (MR Solution, Guildford, Surrey, UK). The scan parameters were as follows: energy, 40 kVp; exposure, 100 ms; current, 1 mA; and stepping angle, 1°. During the imaging procedure the animals were anesthetized with a 3% isoflurane in air and maintained at a temperature of +37 °C. The CT images so obtained were processed using VivoQuant (Invicro, London, UK) software.

### 2.6. Plasma Cytokine Measurement

The blood from individual animals was collected 12 h after SCI into EDTA tubes to obtain plasma. Frozen samples of isolated spinal cord were homogenized (Dounce homogenizer, Thomas Scientific, Swedesboro, NJ, USA) and transferred to 10 volumes (*w*/*w*) of ice-cold PBS supplemented with 0.1% Tween 20 and a protease inhibitor cocktail. The prepared spinal cord homogenate was vortexed for 15 s and subjected to rotator for 5 min at room temperature. Further cell debris was removed by centrifugations at 4 °C (30,000× *g* for 10 min). The supernatant was further used for measurement. Milliplex Rat Cytokine/Chemokine Magnetic Bead Panel (Merck, Rahway, NJ, USA) was used to measure the levels of chemokines and cytokines in plasma and spinal cord extracts. Samples were diluted at 1:4 ratio and further incubated with magnetic beads, washed up, and then incubated with detecting antibodies and SA-PE. The data were obtained using a Luminex 200 analyzer using xPONENT software (Luminex, Austin, TX, USA).

### 2.7. Histological Analysis

Histological studies of rat spinal cord were performed at days 1, 7, 14, 21, 30 and 60, as described previously [12,13]. Briefly, samples of the rat spinal cord encased in bone corresponding to the length of three vertebrae (Th13 was the vertebra of the surgical approach, and Th12 and L1 were the two adjacent vertebrae) were fixed in 10% neutral buffered formalin, rinsed in tap water and processed for decalcification in Trilon B at room temperature for 12–16 days. The biomaterial was oriented for further microtomy in the sagittal planes. Hematoxylin and eosin staining was used for serial 4–5 μm thick paraffin-embedded sections. The sections were examined by the standard light microscopy with an Axio Scope.A1 microscope (Carl Zeiss, Oberkochen, Germany). Photomicrographs of the histological sections were made with an Axiocam 305 color high-speed camera (Carl Zeiss, Oberkochen, Germany).

### 2.8. Statistical Analysis

The statistical analysis was performed by SPSS Statistics 28 software (IBM, Armonk, NY, USA). *p* values less than 0.05 calculated by a Mann–Whitney test were considered as statistically significant.

## 3. Results

### 3.1. Spinal Cord Cryoinjury Model in SD Rats

**Figure 1:**
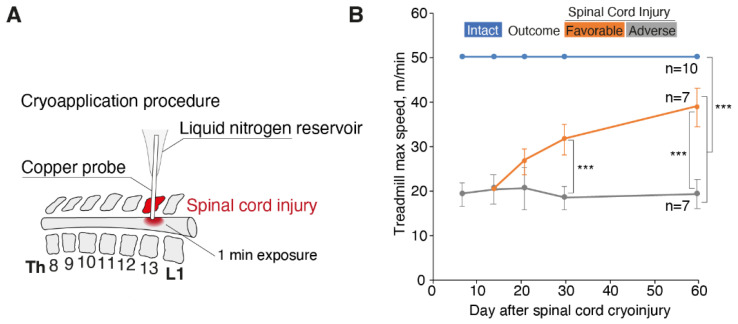
Motor activity of the animals with spinal cord cryoinjury in the groups with favorable and adverse outcomes. (**A**) Spinal cord cryoinjury technique. (**B**) Maximal animal speed (m/min) on a treadmill in groups with favorable and adverse outcomes in comparison with intact animals. Bars represent standard deviation, the *p* < 0.001 (***) values according to the Mann–Whitney test are indicated.

We used a previously elaborated model of spinal cord cryoinjury [12,13] characterized by monoplegia of the hind limb, which can last several months (Figure 1A). Functional tests showed that intact animals easily reached the preset maximum speed of 50 m/min, whereas the animals with spinal cord injury reached a mean speed of 19 m/min. Animals with SCI were divided into two groups with either favorable or adverse outcome according to the dynamics of their recovery from injury (Figure 1B).

### 3.2. Histological Analysis of the Spinal Cord Cryoinjury

**Figure 2:**
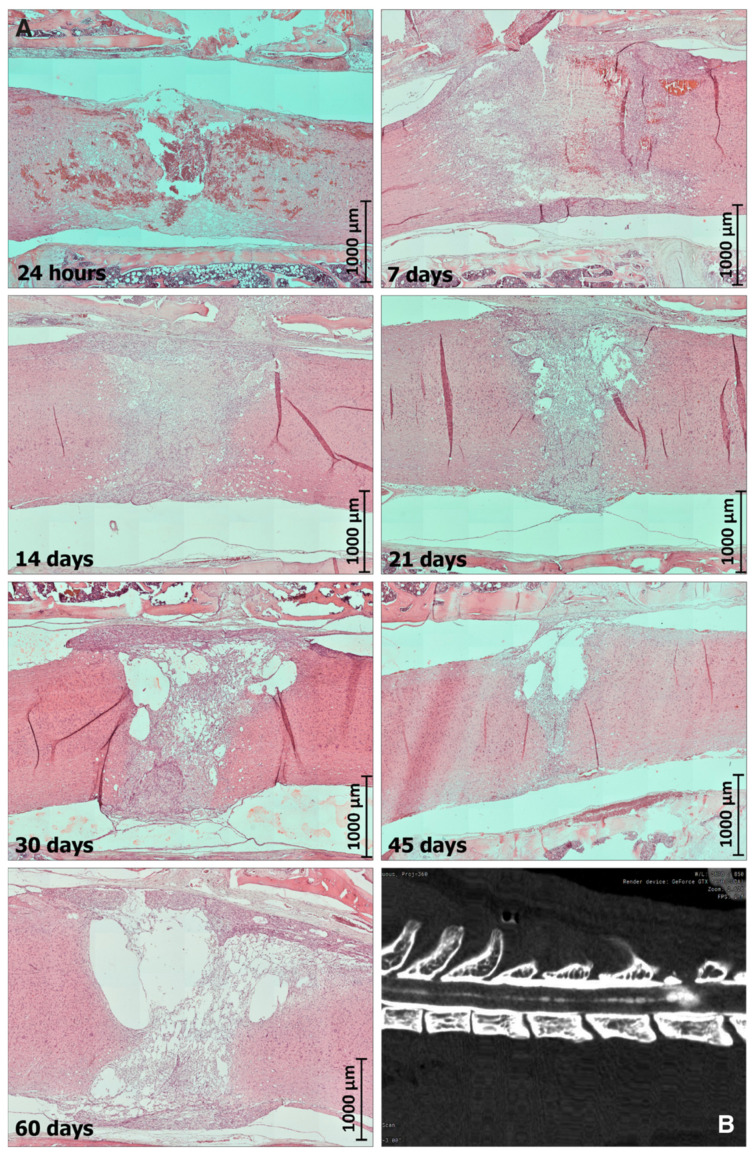
Dynamics of the glial scar formation after spinal cord cryoinjury in SD rats. (**A**) Hematoxylin and eosin staining of a sagittal section of the spinal cord in the vertebral column on day 0, 7, 14, 21, 30 and 60 after the cryoinjury. Scale bars are indicated. (**B**) Contrast visualization of the posttraumatic cyst in thoracic spine.

During the acute period (24 h), there were tissue debris and massive microhemorrhages with a pronounced imbibition of the spinal cord tissues with fresh erythrocytes that could cause the expansion of a necrotic zone during the first days of the postinjury period (Figure 2). On day 7 after the cryoinjury, macrophages were evenly distributed throughout a larger area of the lesion in the spinal cord; their highest concentration was observed at the projection of the spinal gray matter (Figure 2). On day 14 after the cryoinjury, the percentage of macrophages in the total cell population was still decreasing; newly emerged multiple clusters of glial cells were spread unevenly over the lesion. The scar tissue was characterized by a regular vascularization pattern and the blood vessels returned to an ordinary histological organization. By day 14, the mean specific volume of the blood vessels in the lesion increased considerably and reached its specific volume of collagen fibers in the glial scar structure. A pronounced reactivity of astroglia was found in the peripheral portion of the lesion, at the projection of the ventral funiculi of spinal white matter (Figure 2).

In the subchronical period, on day 21, the clusters of glial cells were seen in the peripheral portion of the lesion. The fraction of fibrous tissue component in the structure of gliomesodermal scar was noticeably increased compared to day 14. At the same time, the artificial hollow spaces in the sections were still present (Figure 2). Of note, 3 weeks after the exposure, a trend toward the development of cystic cavities was observed at the site of the cryoapplication. By day 30, multiple macrophages in the focus of the spinal cord defect were still numerous, distributed evenly over the entire area of the formed spinal cord defect, with variations in morphology and size within the same field of view. Numerous large thin-walled racemose cavities, optically “empty” or filled with amorphous eosinophilic components with single macrophages, were typically present in the projection of the gray matter and dorsal cords of the white matter (Figure 2).

At the chronic stage, two months after the cryoinjury, a spinal cord lesion with large cystic cavities containing an amorphous substance was present in all animals (Figure 2). In some animals, racemose cavities were found cranial to the defect, along the spinal (central) canal, indicating impaired liquor dynamics. Thus, by day 60 of the observation period, histological data indicated that glial scar structures were stabilized and a mature fibrous component was formed.

### 3.3. Magnetic Resonance Imaging of the Spinal Cord Cryoinjury

**Figure 3:**
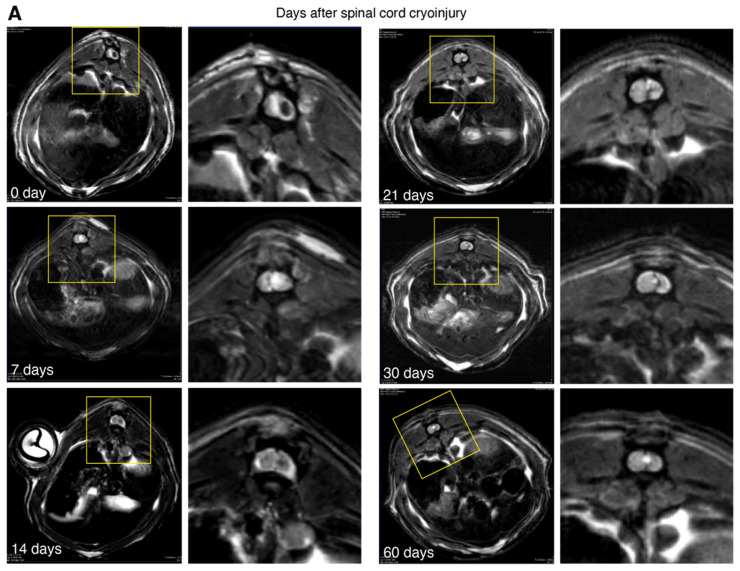

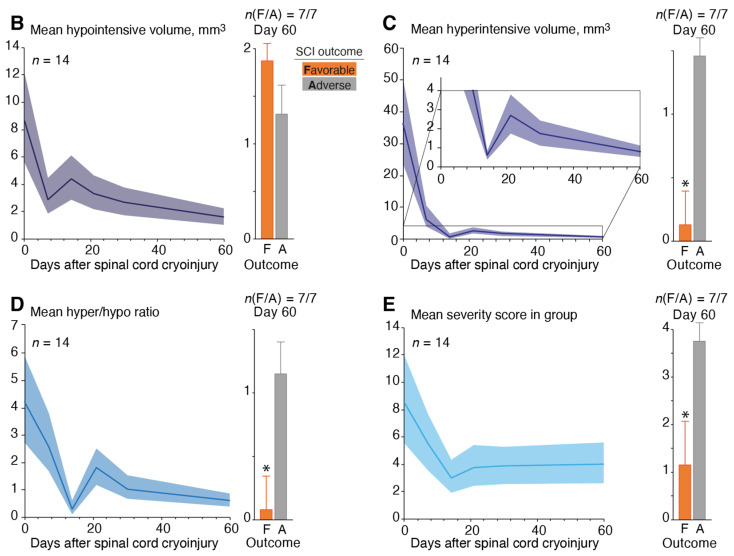
Magnetic resonance imaging of the spinal cord cryoinjury. (**A**) Representative MRI at 0, 7,14, 21, 30 and 60 days. Mean hypointensive (**B**) and hyperintensive (**C**) volume. Mean hyper/hypo ratio (**D**) and mean severity score (**E**). Colored area represents standard deviation. Right bars on each plot demonstrate values at day 60 in groups with favorable (F) and adverse (A) outcomes. Standard deviation and statistically significant difference (*) are shown.

The T1- and T2-weighted MRI images acquired 30 min after the injury (0 day) demonstrated distinct hypointense foci of either round or craniocaudally elongated shape, up to 3.5 × 1.5 mm in size on the right side of the cord, combined with postoperative changes in the soft tissue (Figure 3A). Typically, the hypointense area was linked latero-dorsally with the dura mater as well as with adjacent spinal vessels (a. spinalis dorsalis dextra and v. spinalis ventralis). This area was presumed to be the consequence of acute injury, i.e., hemorrhage or necrosis. An extensive diffuse hyperintense area was seen around it, indicating posttraumatic oedema. Typically, a homogenous dynamic was observed, which was quite prominent in the first hours after injury, and decreased substantially to complete disappearance on day 7, with subsequent reappearance by day 14 as longitudinal ellipsoids of hourglass shape. Subsequently, this area gradually decreased in volume, transforming into characteristic sinuous, branched or “canyon”-like areas, presumably due to glial scar formation.

Additionally, T2-weighted images acquired early after the injury demonstrated extensive diffuse hyperintense areas in the region of interest, presumably representing traumatic edema, which later gradually diminished and mostly disappeared by day 30. In the meantime, new distinct, round-shaped, highly hyperintense structures were detected from day 14 to day 21, contributing to overall the hyperintensive value. These structures represented posttraumatic syringomyelic cysts, which were mainly located within or in close proximity to the central spinal canal.

On day 30, the evolution of hypointense area stopped and gradually decreased in volume while assuming at the same time a tortious aspect. It was possible to trace the presence of the focal hyperintense focus with clear contours in close relationship with the central spinal cord canal. On day 60, an increase in volume of the hyperintense areas was observed indicating the formation of syringomyelic cysts.

Of significance was the fact that at later stages (45–60 days), an evident difference between groups with favorable and adverse outcomes in terms of hyperintensive to hypointensive volume ratio and mean severity score (Figure 3B–E) was observed. Animals from the group with favorable outcomes had no cysts on the MRI images and demonstrated a lower hyperintensive/hypointensive ratio and mean severity score. In the group with adverse outcomes, posttraumatic cysts were clearly visualized, and the mean severity score was significantly higher in comparison with favorable outcomes (3.75 and 1.16, respectively).

### 3.4. Determination of Cytokine Status during Spinal Cord Cryoinjury

Since histological analysis revealed massive infiltration of the neutrophil granulocytes and macrophages into the area of the SCI, we further measured the level of plasma cytokines by a multiplex immunoassay technique in intact animals and 12 h after SCI (Table 2). Each animal with SCI was assigned to either a group with favorable or adverse outcome as described above. Our data suggested that proinflammatory cytokines evidently play an important role in SCI pathogenesis. A statistically significant increase in IL-1α (7.2-fold) was observed 12 h after SCI induction in the group with favorable outcomes in comparison with intact animals, while the levels of IL-1β, TNFα and IL-17A in this group were substantially decreased (2-, 8.9- and 3-fold, respectively). In the group with an adverse SCI outcome, the role of proinflammatory cytokines was completely different. Twelve hours after trauma induction, the plasma levels of IL-1α, IL-1β and TNFα were significantly increased in animals with SCI in comparison with intact animals. The most pronounced drop (107-fold) was observed for IL-1α, whereas the levels of IL-1β and TNFα increased to a lesser extent (13-fold and 2-fold, respectively). IL-6 and IL-17A also tended to increase; however, due to a high variability of the data obtained, this increase was not statistically significant.

**Figure 4:**
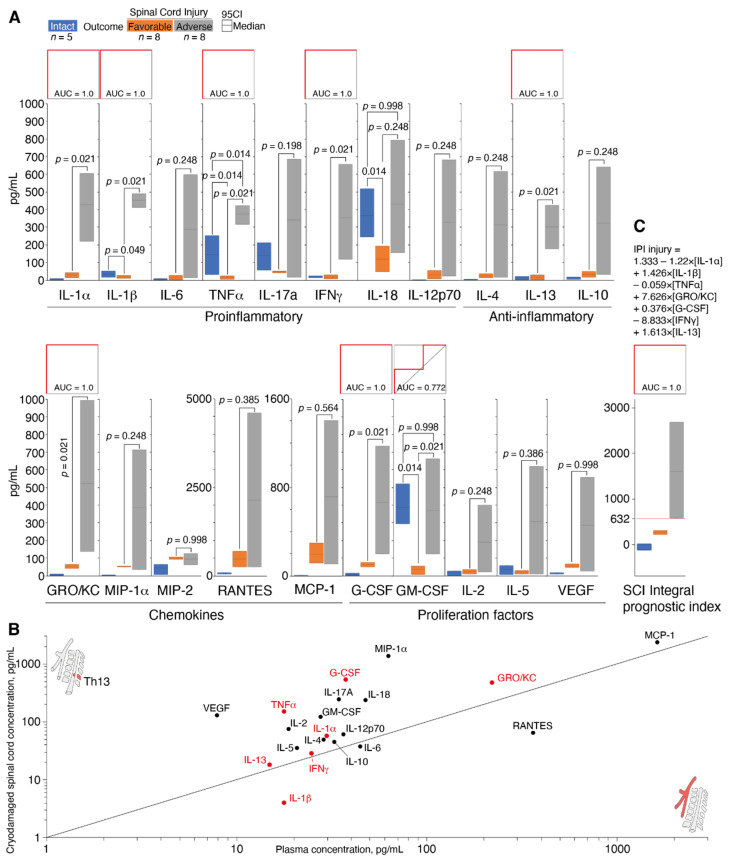
Levels of plasma cytokines and chemokines in the animals with spinal cord cryoinjury in the groups with favorable and adverse outcomes. (**A**) The 95% confidence intervals (CI) for the levels of plasma cytokines and chemokines and their predictive value based on ROC curves. (**B**) Correlation plot of cytokines/chemokines concentration in plasma and injured spinal cord. Factors with ROC = 1 are shown in red. (**C**) The 95% confidence intervals for the integral prognostic index of injury (IPI injury) and its predictive value based on the ROC curve. Bars represent standard 95% CI in groups with favorable (orange) and adverse outcomes (grey) in comparison with intact animals (blue), the *p* values according to the Mann–Whitney test are indicated. Granulocyte colony-stimulating factor (G-CSF), vascular endothelial growth factor (VEGF), granulocyte-macrophage colony-stimulating factor (GM-CSF), keratinocyte chemoattractant (KC), tumor necrosis factor (TNF), interferon gamma (IFNγ), Regulated upon Activation, Normal T Cell Expressed and Presumably Secreted (RANTES/CCL5), monocyte chemoattractant protein 1 (MCP-1/CCL2) and macrophage inflammatory proteins (MIP)—MIP-1a and MIP2.

To determine the diagnostic significance of the proinflammatory cytokines, their 95% confidence intervals were compared, and ROC curves were plotted (Figure 4A). The predictive value of IL-1α, IL-1β, IFNγ and TNFα was high as the AUC had the maximum value of 1.0 (Figure 4A). Our data suggested that plasma levels of IL-1α > 221.5 pg/mL, IL-1β > 413 pg/mL, IFNγ > 118 pg/mL and TNFα > 318 pg/mL predicted an adverse outcome of posttraumatic defect formation.

The level of chemokines as prognostic factors of an SCI adverse outcome was less informative than proinflammatory cytokines. Among all chemokines tested during the study, only GRO/KC demonstrated a significant difference between groups with favorable and adverse outcomes. The level of GRO/KC was 10 times higher in the group with adverse outcomes in comparison with the group with favorable outcomes. The GRO/KC had an absolute predictive value (AUC = 1.0) at concentrations above 137 pg/mL.

Among the growth factors, the colony-stimulating factors for phagocytic cells, namely granulocyte colony-stimulating factor (G-CSF) and granulocyte-macrophage colony-stimulating factor (GM-CSF), had a high predictive value. The level of G-CSF was increased 6.7-fold in the adverse SCI outcome group as compared to the favorable outcome group, and it may be used as a prognostic factor of adverse outcome at levels above 126.5 pg/mL.

Among anti-inflammatory cytokines, only IL-13 had predictive value (Figure 4A). The plasma level of IL-13 increased 18.2-fold in the group with adverse outcomes in comparison with favorable outcome. The predictive value of IL-13 was 1.0 above concentration of 177.5 pg/mL.

Previously it was shown that the levels of MIP-1α, MCP-1 and GRO/KC were increased in the area of injury in a dosed contusion SCI model [14]. We further compared the plasma levels of chemokines and cytokines with their release in the cryodamaged spinal cord (Figure 4B). Our results fully correlated with data reported by Mukhamedshina et al. [14] as MIP-1α, MCP-1 and GRO/KC together with G-CSF were the most abundant factors in the area of spinal cord injury 12 h post cryoapplication. The correlation plot of cytokines/chemokines concentration in plasma and injured spinal cord shown on Figure 4B suggested that the site of the cryoapplication was the main source of the cytokine storm observed in plasma.

Summarizing, seven cytokines and chemokines (IL-1α, IL-1β, TNFα, GRO/KC, G-CSF, IFNγ, IL-13) may be considered as prognostically significant molecules. To rule out the possibility of random individual deviations in one or more of these cytokines affecting the prognosis of the spinal cord cryoinjury outcome, we generated an integral prognostic index (IPI) in the form of a linear regression equation (Figure 4B). The IPI fully preserved the maximal predictive value of its internal variables and at the same time excluded random deviations of the individual cytokines. Due to these features, an IPI at values above 632 may be suggested as a reliable criterion for an adverse outcome of a spinal cord cryoinjury.

## 4. Discussion

In the last two decades, it has been suggested that traumatized spinal cord axons attempt to regrow [15]. However, the process of their spontaneous regeneration is hampered by several local posttraumatic reaction phenomena, which ultimately lead to glial scarring and cyst formation. These factors evidently act against spontaneous neuroregeneration [6]. A better insight of this highly complex process could lead to a better understanding of the pathophysiology of early spinal posttraumatic events and could be helpful for the development of more effective treatment protocols. In this study we used a recently proposed rat SCI model accomplished by a controlled cryoapplication. Our technique, unlike other models of traumatic spinal cord injury, consistently reproduces glial scarring and the posttraumatic formation of cysts [13], which both play a fundamentally negative role against potential neuroregeneration [15,16,17].

We found that the plasma levels of almost all cytokines were significantly upregulated following traumatic spinal cord injury. We focused on cytokine production during the early acute stage of the spinal cord injury [18,19], since on the second day following SCI, the release of cytokines by bone marrow cells decreases [20]. Importantly, groups of experimental animals with favorable and adverse outcomes, which initially were formed according to nonsubjective neurophysiologic treadmill test, demonstrated a dramatic difference in the severity of the plasma cytokine storm after 12 h following SCI.

Previously it was shown that surgery procedure accompanied by exposure to isoflurane, oppositely, decreased levels of serum cytokines 6 h later [21] except those of GRO/KC, MCP-1, IL-18, M-CSF and MIP-1α. Nonetheless, the upregulation of these factors reached 2–3 fold, whereas in our study, the concentration of cytokines and chemokines in the plasma following experimental SCI increased up to two orders of magnitude in comparison with intact animals. Therefore, the enhanced release of cytokines and chemokines was mainly caused by a pathological process in spinal cord, but not surgical intervention or anesthesia itself.

Proinflammatory cytokines such as IL-1, IL-6 and TNFα play a principal pathogenic role in spinal cord injuries [22,23]. Cytokines IL-1 (IL-1α and IL-1β) and TNFα were identified in our study as prognostically relevant factors. Our observations may be linked with the ability of these cytokines to damage and destroy the neurons when their concentration is above a certain threshold. Importantly, decreased serum level of TNFα and Il-1β in SCI patients after 9 h following injury correlated with the American Spinal Injury Association’s (ASIA) impairment scale (AIS) improvement [24]. The upregulation of the IL-1 in the bone marrow leads to an increase of vascular permeability and attracts lymphocytes [18]. TNFα is a cytokine which is activated in neurons, glia and endothelial cells following SCI [25] and attracts neutrophils to the damaged area through the induction of adhesion molecules [26]. An increase in the level of TNFα leads to a change of endothelial cell permeability, which, in turn, results in the impairment of blood–marrow barrier. TNFα may cause the death of the oligodendrocytes [27] and result in demyelination [28]. On the other hand, proinflammatory cytokine IL-6, known for its beneficial effect on axon regeneration and gliosis [29,30], was not considered in the integral prognostic index.

Among chemokines, only GRO/KC was identified in the current study as a prognostically relevant factor. An enhanced level of this chemokine in the serum of SCI patients was associated with injury at the cervical region [31] and peaked on day 7 after trauma [32]. An increase of this chemokine with its neuroprotective function and ability to cause chemotaxis of neutrophils on the third day of experimental SCI was shown previously [14]. One may suppose that a significant increase in the level of this cytokine already at 12 h after trauma induction may have an adverse effect on the course of the regenerative process following SCI due to the excessive recruitment of neutrophils to the damaged area at such an early stage of the pathological process triggered by SCI.

G-CSF, which was also upregulated in the adverse outcome group, is known to exert marked anti-inflammatory, antiapoptotic, antioxidant, myelin-protective and regenerative effects on axons in acute, subacute and chronic CNS damages. Additional effects of this cytokine result in the induction of angiogenesis and neurogenesis as well as in the recruitment of bone marrow stem cells to CNS. Due to these effects, G-CSF was recently suggested for treatment of acute and chronic traumatic spinal cord injury [33]. Sanli et al. [34] previously showed that a systemic administration of G-CSF was associated with an increase in the number of neutrophils in the peripheral blood and a concomitant decrease of this number in damaged tissue. The authors suggested that G-CSF reduced the synthesis of potentially toxic proinflammatory cytokines in SCI and also decreased lipid peroxidation with their effects on fluidity and other characteristics of cell membrane which was reported following treatment with G-CSF.

The role of IFNγ, which was associated with adverse outcomes of spinal cord cryoinjury due the process of large cysts formation in a damaged area, in SCI is still controversial. Despite the involvement of IFNγ in the inflammatory reaction, IFNγ seems to improve the outcome of traumatic spinal cord injury, although positive and negative results were obtained utilizing genetic models [35]. A recent study demonstrated that the level of IFNγ was still 52-fold increased in the serum of patients with SCI two weeks after injury [31].

Finally, we detected an increased plasma level of IL-13 as a marker of adverse outcomes of SCI. Despite the induction of the expression of anti-inflammatory markers in microglia and macrophages following SCI, IL-13, contrary to IL-4, did not mediate the functional regeneration of bone marrow [36], which is in accordance with our findings.

Summarizing, regardless of current discrepancies in the evaluation of a pathogenetic role of cytokines and chemokines at early stages of SCI, our data, expressed mathematically in the form of an integral prognostic index, had an unequivocal and statistically confirmed value as a prognostic marker of adverse outcome of posttraumatic defect formation after spinal cord cryoinjury.

MRI imaging of the spinal cord in vivo provides a homogeneous and reliable visual control of the dynamics of posttraumatic processes and clearly shows the extent of early damage, the formation of glial scars, and the development of posttraumatic syringomyelic cysts. Previous studies emphasized the important role of MRI in the monitoring of the evolution of posttraumatic processes in SCI [37,38]. These reports usually utilized nonconventional MRI techniques, such as DATE and, more recently, DTI [39,40]. In the present study, we purposely used standard T1- and T2-weighted imaging protocols to shorten data acquisition time, thus decreasing the risk of animal mortality during the experiments due to a reduced time of anesthesia.

Here, we proposed a 10-grade scoring scale, which could objectively estimate the severity of SCI according to the MRI results. Such a quantitative analysis is especially important in the case of small experimental animals. MRI may be used for the evaluation of the efficacy of perspective therapeutic protocols without the necessity for more invasive as well as postmortem diagnostics. Our MRI data were strictly consistent with the levels of plasma cytokines, treadmill test and histology analysis. We found that the areas of cryodestruction in serial sagittal sections were transmural in nature and were orientated in the dorsoventral direction of the spinal cord throughout its entire structure. Thus, the manifestation of small racemose cavities formation at the site of the cryoapplication could be observed from day 14. By day 30–60, we should expect the development of the fusion of the racemose cavities, with their enlargement due to impaired SCF dynamics. CT myelography demonstrated that the contrast agent still reached the posttraumatic cyst a few hours after administration. We believe that future studies should confirm our observation which might indicate that CSF pathways can be used for the local delivery of bioactive agents into posttraumatic syringomyelic cysts. In the future, our MRI and immunological findings may result in specific algorithms to adopt therapeutic strategies that prevent inflammatory damages in spinal cord and help to restore its integrity.

## 5. Conclusions

We demonstrated that quantitative MRI data together with a determination of the levels of plasma cytokines directly correlated with clinical outcome in this spinal cord cryoinjury model. The suggestions coming from the present study may be translated to the clinical practice in patients with SCI. Our study delivers novel noninvasive and intravital methods for the evaluation of the efficacy of therapeutic protocols for SCI treatment, which may be tested in preclinical trials in the nearest future.

## Figures and Tables

**Table 1:** Visual assessment scoring of severity of spinal cord injury according to MRI data.

Criterion	Significant (3 Points)	Moderate (2 Points)	Mild (1 Point)	None (0 Points)
Hyperintense area volume, mm^3^	>10	5–10	<5	Not defined
Hypointense area volume, mm^3^	>5	2–5	<2	Not defined
VHyper/VHypo ratio	>3	2–3	1–2	0–1
Clear signs of syringomyelia cyst	Adds 1 point to final score			

**Table 2:** Cytokine profile of blood plasma from animals with spinal cord cryoinjury.

Cytokine/Chemokine Group		Median [Minimum; Maximum] of Plasma Level (pg/mL)			*p* Value, Mann–Whitney Test		
		Intact *n* = 5	12 h after SCI		Favorable Outcome vs. Intact	Adverse Outcome vs. Intact	Favorable vs. Adverse Outcome
			Favorable Outcome *n* = 8	Adverse Outcome *n* = 8			
Proinflammatory cytokines	IL-1α	4 [1.5; 7.5]	28.8 [14.5; 42]	427.5 [221.5; 605.5]	0.014	0.014	0.021
	IL-1β	33.5 [17.5; 50.5]	17 [10; 25]	451.5 [413; 491]	0.049	0.014	0.021
	IL-6	5.5 [2; 9]	15 [2; 27]	288 [15; 598]	0.389	0.014	0.248
	TNFα	142.5 [32; 251.5]	16 [6; 23]	375 [318; 422]	0.014	0.014	0.021
	IL-17A	140.5 [58.5; 210.5]	46.5 [43; 52.5]	341.5 [18; 685.5]	0.014	0.048	0.198
	IFNγ	20 [13; 24]	18.5 [7; 30.5]	355 [118; 658]	0.998	0.014	0.021
	IL-12p70	0.5 [0; 1]	30 [7; 53]	330.5 [23; 682]	0.014	0.014	0.248
	IL-18	366 [247; 518]	118.8 [49.5; 192]	430.5 [157; 794]	0.014	0.998	0.248
Chemokines	GRO/KC	5.5 [2.5; 11.5]	52.5 [42; 62]	525.5 [137; 993.5]	0.014	0.014	0.021
	MCP-1	1.2 [0.9; 2]	196 [114; 294]	715.5 [105.5; 1402.5]	0.014	0.014	0.564
	MIP-1α	3 [1; 5]	52.8 [50.5; 55]	386 [35; 712.5]	0.014	0.014	0.248
	MIP-2	44 [6.5; 64.5]	99.5 [95; 108]	94 [63; 124]	0.014	0.027	0.998
	RANTES	66.5 [47; 89.5]	467.5 [249; 688]	2147.5 [250; 4598]	0.014	0.014	0.386
Proliferation factors	G-CSF	7 [3; 13]	62 [51; 75]	416 [126.5; 734]	0.014	0.014	0.021
	GM-CSF	389 [294; 522]	35 [8; 53]	369 [124; 664]	0.014	0.998	0.021
	IL-2	16.5 [1.5; 26]	22.5 [11; 34.5]	191 [23.5; 401]	0.327	0.049	0.248
	IL-5	34.5 [6.5; 57.5]	21 [10; 30.5]	308 [12; 619]	0.623	0.325	0.386
	VEGF	12 [10; 17]	58.5 [48.5; 65]	284.5 [26; 559]	0.014	0.014	0.998
Anti-inflammatory cytokines	IL-4	1.5 [0.5; 3.5]	23 [12.5; 35]	314.5 [18; 618]	0.014	0.014	0.248
	IL-13	14.5 [2.5; 20.5]	16.5 [3; 29]	301 [177.5; 424]	0.462	0.014	0.021
	IL-10	6 [4; 16]	33 [16; 49]	324 [32; 640]	0.019	0.014	0.248

## Data Availability

All data generated or analyzed during this study are included in the article.
